# Integrated pan‐cancer of *AURKA* expression and drug sensitivity analysis reveals increased expression of *AURKA* is responsible for drug resistance

**DOI:** 10.1002/cam4.4161

**Published:** 2021-08-01

**Authors:** Noushin Miralaei, Ahmad Majd, Kamran Ghaedi, Maryam Peymani, Masoomeh Safaei

**Affiliations:** ^1^ Department of Biology Tehran North Branch Islamic Azad University Tehran Iran; ^2^ Department of Cell and Molecular Biology and Microbiology Faculty of Biological Science and Technology University of Isfahan Isfahan Iran; ^3^ Department of Biology Faculty of Basic Sciences Shahrekord Branch Islamic Azad University Shahrekord Iran; ^4^ Department of Pathology Cancer Institute Imam Khomeini Hospital Complex Tehran University of Medical Sciences Tehran Iran

**Keywords:** *AURKA*, biomarker, cancer, drug resistance, prognosis

## Abstract

**Introduction:**

The *AURKA* gene encodes a protein kinase involved in cell cycle regulation and plays an oncogenic role in many cancers. The main objective of this study is to analyze *AURKA* expression in 13 common cancers and its role in prognostic and drug resistance.

**Method:**

Using the cancer genome atlas (TCGA) as well as CCLE and GDSC data, the level of *AURKA* gene expression and its role in prognosis and its association with drug resistance were evaluated, respectively. In addition, the expression level of *AURKA* was assessed in colorectal cancer (CRC) and gastric cancer (GC) samples. Besides, using Gene Expression Omnibus (GEO) data, drugs that could affect the expression level of this gene were also identified.

**Results:**

The results indicated that the expression level of *AURKA* gene in 13 common cancers increased significantly compared to normal samples or it survived poorly (HR >1, *p* < 0.01) in lung, prostate, kidney, bladder, and uterine cancers. Also, the gene expression data showed increased expression in CRC and GC samples compared to normal ones. The level of *AURKA* was significantly associated with the resistance to SB 505124, NU‐7441, and irinotecan drugs (*p* < 0.01). Eventually, GEO data showed that JQ1, actinomycin D1, and camptothecin could reduce the expression of *AURKA* gene in different cancer cell lines (logFC < 1, *p* < 0.01).

**Conclusion:**

Increased expression of *AURKA* is observed in prevalent cancers and associated with poor prognostic and the development of drug resistance. In addition, some chemotherapy drugs can reduce the expression of this gene.

## Graphical abstract

The expression level of AURKA gene in 13 common cancers increased significantly compared to normal. The level of AURKA was significantly associated with resistance to SB 505124, NU‐7441, and irinotecan drugs (*p* < 0.01). Actinomycin D1 and camptothecin could reduce the expression of AURKA gene in different cancer cell lines based on GEO data.

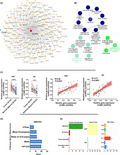

## 1 INTRODUCTION

One of the biggest health problems in the world is cancer, and based on estimates from the world health organization (WHO), cancer is the first or second cause of death before the age of 70 years.^1^, ^2^, ^3^ Cancers can have many forms based on the location and cell of origin, and they are known to be genomic diseases. Scientists are faced with variety of cancer challenges; however, tumor heterogeneity, which has the potential to be seen in the expression level of genes, mutations, epigenetic, and microenvironment, has been on the top of attentions.^4^ Therefore, what can help controlling, treating, and diagnosing cancers is identifying key genes or the vital pathways among all sorts of cancers.^5^, ^6^

*AURKA* is a serine/threonine kinase that is crucial in controlling of mitosis progression, centrosome maturation/separation, and mitotic spindle function.^7^, ^8^, ^9^ The high expression of *AURKA* has been seen in many sorts of cancers such as ovarian, liver, and colorectal ones making some oncogenic factors like c‐MYC, NF‐kB, and β‐catenin active, and it could lead to chromosomal instability.^10^, ^11^ Besides, recent studies have mentioned that *AURKA* probably takes part in cancer development and progression as well as tumorigenesis.^12^, ^13^ In fact, the high level of *AURKA* leads to blockage of TP53 as a tumor suppressor by phosphorylation at Ser215 and Ser315.^14^, ^15^, ^16^ Actually, the indirect relation between the expression level of TP53 and *AURKA* can promote carcinogenesis and progression as a negative feedback.^17^, ^18^ Moreover, previous studies have indicated that the overexpression of *AURKA* in some cancers such as stomach, bladder, and colorectal ones are in inverse relation to disease prognosis.^19^, ^20^

Recent studies have indicated that *AURKA* can be an excellent candidate for kinases inhibitors.^21^, ^22^ Since the overexpression of *AURKA* could stop apoptosis, and promote cancer cell proliferation, the *AURKA* inhibitors (AKIs) have the ability to inhibit the expression of this gene so that the cancer cell would cease to spread and migrate.^23^ As various studies have suggested that, some AKIs have been utilized in preclinical and clinical studies.^17^ Some SNP polymorphisms could increase the risk of cancer in individuals such as the *AURKA* rs2273535 polymorphism in breast cancer.^24^ The expression level of *AURKA* could be utilized in many cancers as a biomarker that might detect some cancers at the beginning levels.^25^

Many studies have shown that the *AURKA* gene has an oncogenic role in some cancers, but its expression in some cancers remains unknown. Also, the relation between this gene and drug sensitivity and resistance has not been evaluated, and drugs that affect the expression of this gene are less known. In this study, using the cancer genome atlas (TCGA) data as well as colorectal cancer (CRC) and GC samples, *AURKA* expression levels were examined in 13 common cancers. The role of this gene in resistance, drug sensitivity, and drugs affecting was also investigated using PharmacoDB and GEO data. The results revealed that the *AURKA* expression level in all prevalent cancers increased significantly compared to normal samples, and that it was associated with a poor prognosis in part of cancers. Besides, drugs affecting the expression of this gene were evaluated and can be used in samples with high levels of *AURKA* expression.

## 2 MATERIALS AND METHODS

### 2.1 Data acquisition

To analyze the expression level, mutation, and the relation of *AURKA* with clinical information, TCGA data were utilized. Data processing was performed in accordance with human rights protection and access to TCGA data policies. Based on Table 1, the 13 cancers RNA‐seq data extracted from TCGA in HTseq‐count format including tumor and normal samples. The TCGAbiolinks packages were utilized to process and prepare.^26^ The data were normalized by edgeR and limma (Voom method) packages which are able to trim some genes with a low level of expression, and afterward, the expression level of each gene was brought into log2.^27^ These data were implemented to analyze the expression level of genes, to correlate between many items, and to associate in gene expression and prognostic. DNA‐Seq data from TCGA were used to evaluate the mutation and the type of it in *AURKA*. In this regard, MAF data for all 13 common cancers were downloaded with Pipeline Mutect2.^28^ The maftools package was used to display and calculate the type and frequency of mutations in the *AURKA* gene.^29^

**TABLE 1:** Information about 13 common cancers based on the TCGA database

Project ID	Cancer name	Number of normal samples	Number of tumor samples
BLCA	Bladder urothelial carcinoma	19	414
BRCA	Breast invasive carcinoma	113	1109
COAD	Colon adenocarcinoma	41	480
HNSC	Head and neck squamous cell carcinoma	44	502
LIHC	Liver hepatocellular carcinoma	50	374
KIRP	Kidney renal papillary cell carcinoma	32	289
KIRC	Kidney renal clear cell carcinoma	72	539
KICH	Kidney chromophobe	24	65
LUSC	Lung squamous cell carcinoma	42	502
LUAD	Lung adenocarcinoma	59	535
STAD	Stomach adenocarcinoma	32	375
PRAD	Prostate adenocarcinoma	52	499
UCEC	Uterine corpus endometrial carcinoma	35	552

### 2.2 Prognosis assessment

TCGA clinical data for 13 common cancers were utilized to assess the relation of the expression of *AURKA* with the prognosis. For this purpose, the latest update of clinical data for each cancer sample was downloaded by the TCGAbiolinks package. At first, in order to trim the clinical data, NA data were eliminated. Then the patients whose days of life were 1 or 0 were removed. At the beginning, to assess the prognosis, normal samples were omitted from the expression matrix, and the data were taken in scale mode. In the next step, clinical data were added for each sample and cox regression test was performed to evaluate the association between *AURKA* expression and patients’ prognosis. Finally, Kaplan–Meier test was performed to approve the data.

### 2.3 Expression network and signal pathway, identification of drug resistance and sensitivities

Using the expression network and GEO (gene expression omnibus) data, the pathway that the *AURKA* gene can have an activity on is recognized. For this purpose, all the genes that were commonly expressed in 13 common cancers were identified, and all data were integrated. The SVA package was applied to remove Bach effects from data integration.^30^ Finally, the correlation test was used for *AURKA* expression and all genes. Next, the genes with a correlation coefficient of more than 0.7 (*R* > 0.7) and a significance of less than 0.01 (*p* < 0.01) were selected for expression network and to identify signaling pathway. Also, the ClueGo application in Cytoscape was implemented for data enrichment and drawing cross talk between identified pathways based on Reactome (https://reactome.org) database. CCLE data (https://portals.broadinstitute.org/ccle) and GDSC database (https://www.cancerrxgene.org) for checking the role of *AURKA* in drug resistance, sensitivity, and susceptibility were used by PharmacoGx package. Meanwhile, processed data from both databases were downloaded and studied to examine the correlation of *AURKA* expression and IC50 of different drugs. Besides, the GEO data were implemented to verify the expression network data and to recognize the drugs that are able to lower the level of the *AURKA* in all types of cancers. To achieve this objective, using the keywords *AURKA*, cancer, and treatment, the approved studies were selected. Then, raw data were downloaded and initial preprocessing including background correction, data normalization, and data log2 transformation were performed.

### 2.4 Sample collection, RNA extraction, cDNA synthesis, and RT‐qPCR

This study was approved by the Biomedical Ethics Committee of the Islamic Azad University of Tehran North with the Ethics Code of IR.IAU.TNB.REC.1400.005. The CRC samples including 30 tumor samples and 30 adjacent normal samples, and also gastric cancer samples including 23 tumor samples and 23 adjacent normal samples were obtained from the Tumor Bank of Iran. All samples were approved by a professional pathologist and collected with the patient's consent, and they were kept in liquid nitrogen before use. Clinical information of these samples was shown in Table 2. For total RNA extraction from samples, at first, these tissues were washed three times with PBS to remove contamination as well as necrotic cells. Then RNA extraction was performed with TRIzol (Sigma‐Aldrich) kits, according to the manufacturer's instruction. The extracted RNA for each tissue was treated with *DNase* I to eliminate possible DNA contamination. After that, cDNA synthesis was performed by cDNA synthesis kit (Yekta Tajhiz, Iran) based on original protocol for all samples. The *AURKA* and *CCNB1*‐specific primers were designed by NCBI (https://www.ncbi.nlm.nih.gov/tools/primer‐blast) which these sequences are F: 5′‐TGTGCCTTAACCTCCCTATTC‐3′ and R: 5′‐AACCTTGCCTCCAGATTATGA‐3′ for *AURKA* and F: 5′‐TGCAGGCCAAAATGCCTATG‐3′ and R: 5′‐ACCAAAATAGGCTCAGGCGA‐3′ for *CCNB1*. To assess the level of expression, RT‐qPCR was utilized with using SYBR Green PCR Master Mix (Yekta Tajhiz), 10 pmol/µl of each primer, and 50 ng cDNA in a final volume of 20 µl for each reaction. *GAPDH* (F: 5′‐TGCCGCCTGGAGAAACC‐3′, R: 5′‐TGAAGTCGCAGGAGACAACC‐3′) was considered as an internal control. All measurements were carried out in triplicate and data were analyzed by ΔCt method.^31^

**TABLE 2:** Clinical information for CRC and GC samples

Cancer name	Characteristic	Number (*N* = 30)
CRC	Age	
	<50	9
	>50	21
	Gender	
	Male	18
	Female	12
	TNM stage	
	I	5
	II	11
	III	9
	IV	5
	Tumor size (cm)	
	<5	12
	>5	18
GC	Age	
	<50	8
	>50	15
	Gender	
	Male	9
	Female	14
	TNM stage	
	I	3
	II	9
	III	6
	IV	5
	Tumor size (cm)	
	<5	16
	>5	7

### 2.5 Statistics and software

All gene expression level data were performed in three replications and displayed as ±SD, and Wilcoxon test was used to evaluate the significance of tumor samples compared to normal ones. Mann–Whitney test was employed to evaluate the level of TCGA data expression. In addition, Pearson correlation test was used to identify the correlation of genes with *AURKA*. Log‐rank test was employed to make an assessment of the association of *AURKA* expression with patients’ prognosis. Significance level in all statistical tests of this study was considered less than 0.01 (*p* < 0.01). In this study, Cytoscape (v 3.7), R programming language (V 4.2.1), and GraphPad Prism (v 3.8) were used.

## 3 RESULTS

### 3.1 Pan‐cancer: Increased *AURKA* expression level in common cancers

**FIGURE 1:**
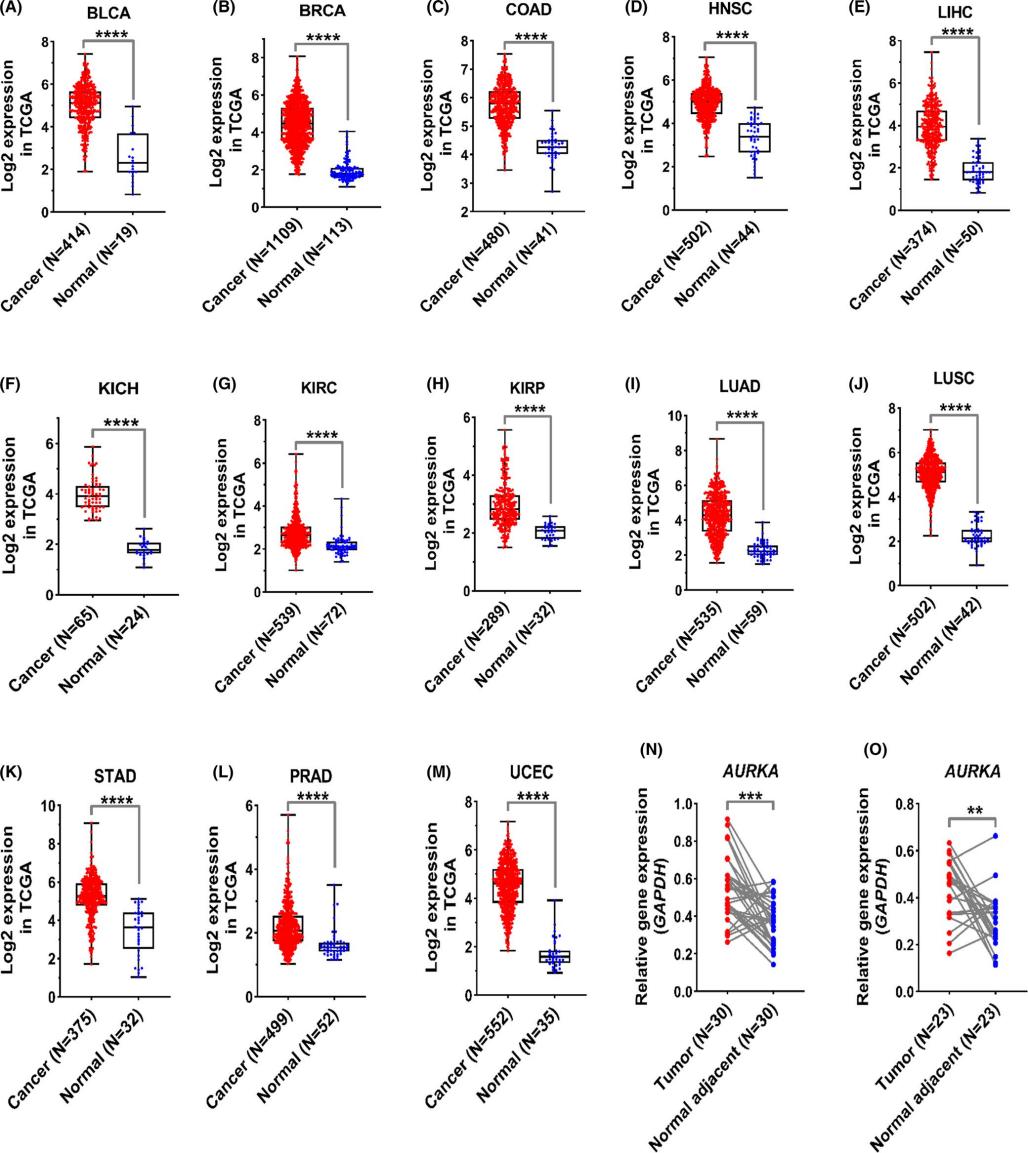
*AURKA* indicated significant upregulation in prevalent cancers compared to normal tissue. (A–M) The expression levels of *AURKA* in 13 prevalent cancers are shown based on TCGA data. The normalized data and logarithmic scale based on 2 were used to draw graphs. (N–O) RT‐qPCR data are shown for CRC and GC specimens. Relative expression levels were shown according to 2^−Δct^. Data were presented as means ± SD of three independent replicates of experiments (****p < 0.0001, ***p < 0.001, **p < 0.01)

**FIGURE 2:**
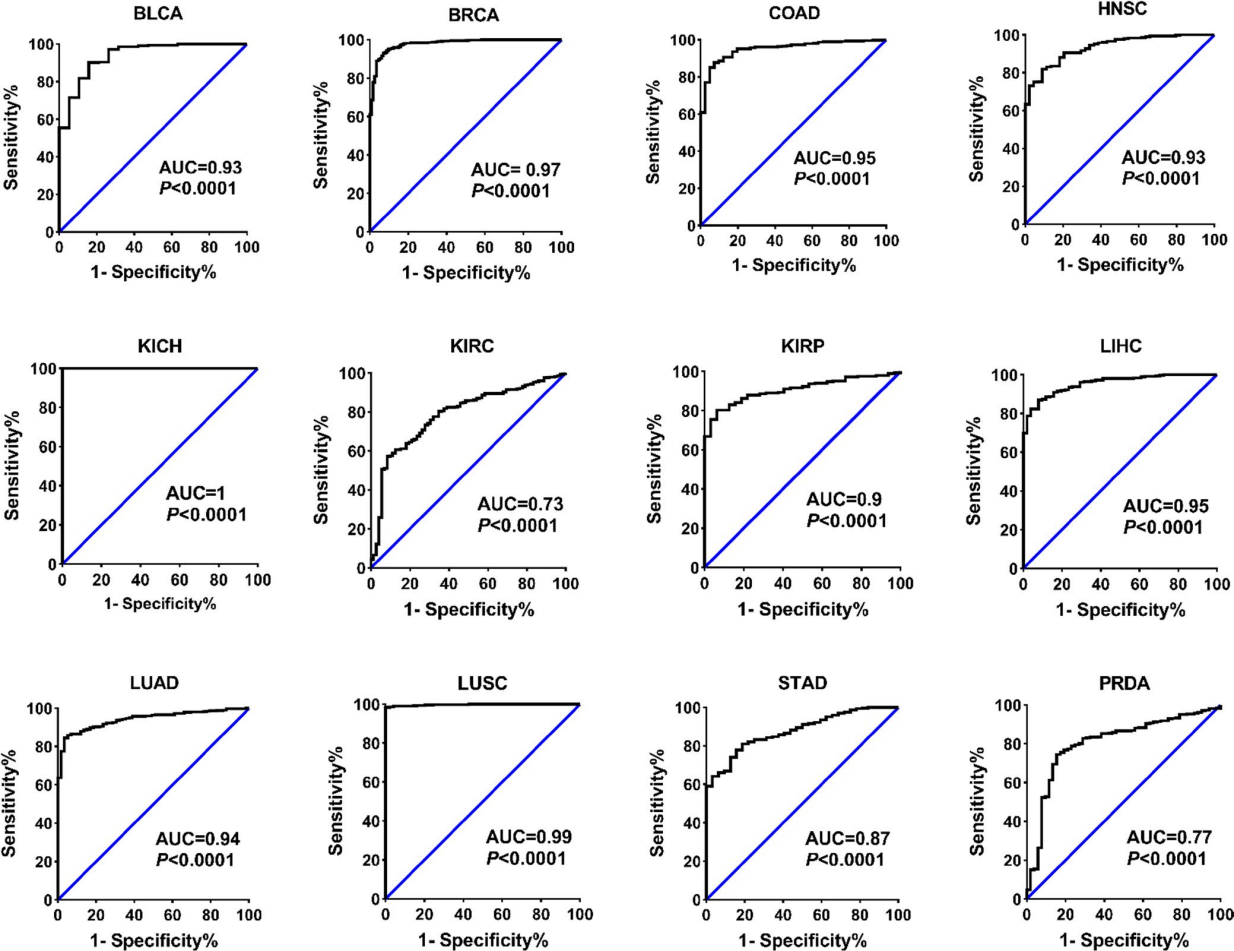
*AURKA* expression level can be recognized as an excellent biomarker for detecting cancer specimens. The *AURKA* level in normal and cancerous samples was used for ROC analysis based on TCGA data. AUC greater than 0.9 is known as excellent biomarkers

Since the expression level of the *AURKA* gene has not been exactly determined in some types of cancers yet, the RNA‐seq data in the TCGA database were employed to evaluate the expression of *AURKA* in the 13 most prevalent cancers. The *AURKA* expression levels in all common cancers including bladder (BLCA), breast (BRCA), colorectal (COAD), head and neck (HNSC), liver (LIHC), some types of kidney (KIRP, KIRC, and KICH), various types of lung (LUSC and LUAD), gastric (STAD), prostate (PRAD), and uterine (UCEC) increased significantly compared to normal samples of each cancer (Figure 1A–M, *p* < 0.001). Furthermore, the similar results obtained when the expression level of AURKA was assessed in the pair samples (Figure S1, *p* < 0.001). The evaluation of *AURKA* transcription level in CRC and GC samples using RT‐qPCR was used to confirm the TCGA data. The results showed that the expression level of the *AURKA* in CRC (Figure 1N) and GC (Figure 1O) samples doubled compared to adjacent normal samples (*p* < 0.01). Furthermore, ROC diagrams for this gene revealed that *AURKA* expression level could be an excellent biomarker for separating cancer cells from normal cells (Figure 2, *p* < 0.001). These results suggested that this gene took part in the onset and progression of prevalent cancer and might be a suitable therapeutic target for many types of cancers.

### 3.2 Involvement of *AURKA* in cell cycle signaling and TP53 pathways in prevalent cancers

**FIGURE 3:**
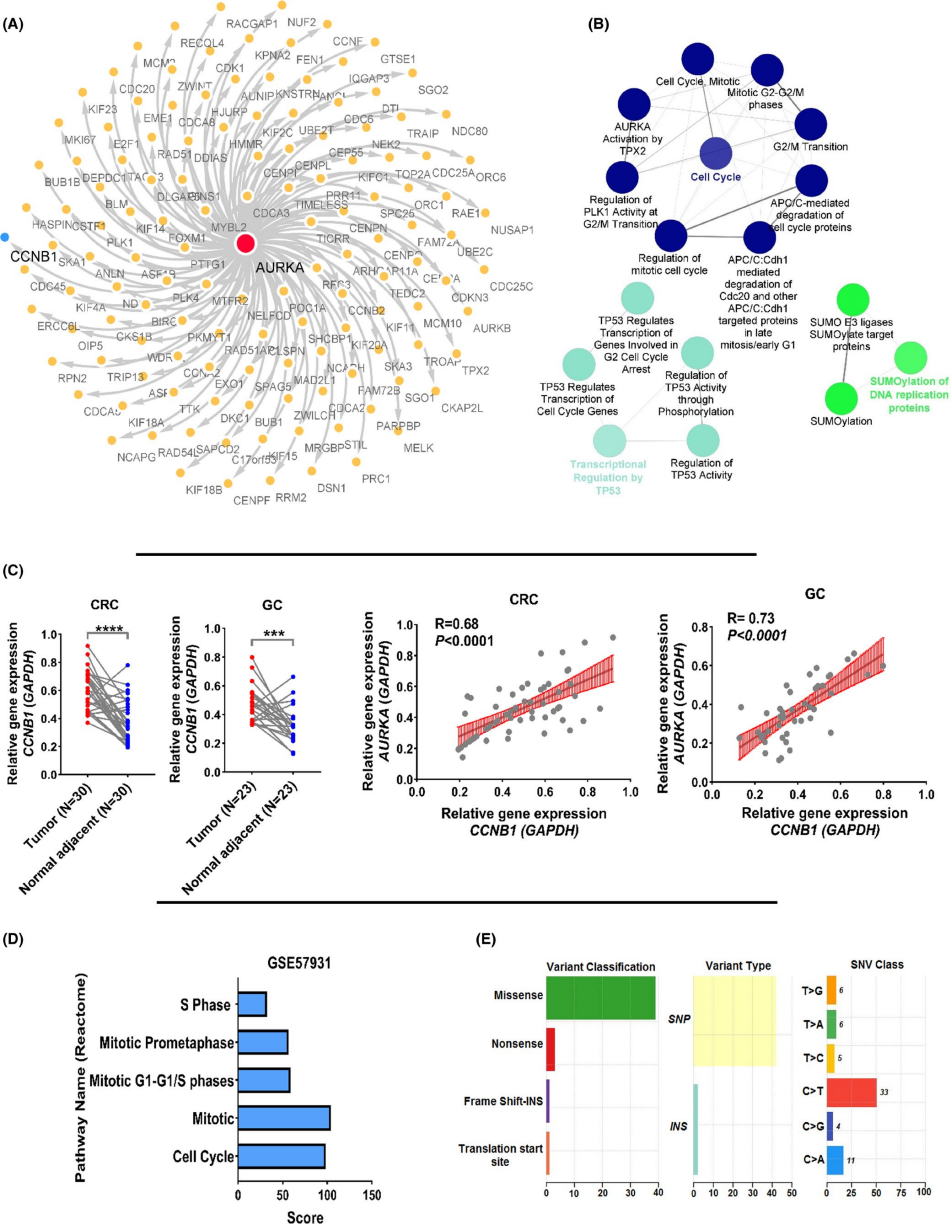
*AURKA* is involved in the cell cycle and tp53 signal pathway. (A) The co‐expression network is shown for all genes that have significant correlation with *AURKA*. (B) The enrichment results shown for all genes in co‐expression network. ClueGo application in Cytoscape was used for enrichment based on Reactome database. (C) *CCNB1* expression is shown in CRC and GC samples compared to normal adjacent samples. Also, the co‐expression between *AURKA* and *CCNB1* levels in CRC and GC samples is indicated. (D) The enrichment results of genes affected by changes in *AURKA* expression are shown in GSE57931. (E) The frequency and different types of *AURKA* gene mutations are shown for all 13 common cancers based on TCGA data

A co‐expression network was used to identify the pathways in which *AURKA* could be involved. All available RNA‐seq data for all 13 common cancers (TCGA data) were merged to achieve the expression of 21,543 genes in 6850 cancers and normal samples. A correlation test was taken between *AURKA* expression level and all genes, and *AURKA*‐related genes were displayed in Figure 3A (*R* > 0.7, *p* < 0.01). Based on gene enrichment results, the *AURKA* gene was associated with genes that regulate the cell cycle pathway as well as the p53 pathway (Figure 3B). To confirm the obtained outcomes from the co‐expression network, the expression level of *CCNB1* that it was in the co‐expression network (Figure 3A, blue node) and involved in the cell cycle and p53 pathways was evaluated in CRC and GC samples. The results indicated that the level of *CCNB1* was upregulated in CRC and GC samples compared to normal adjacent samples (Figure 3C). Moreover, the correlation test results between the levels of *AURKA* with *CCNB1* indicated the strong and significant association in all samples (Figure 3C). In fact, mentioned results confirm the obtained co‐expression network. Also, the study with access number GSE57931 was used to verify the results. This study showed the overexpression of *AURKA* in MCF‐10A cancer cell line, and the change of transcriptome have been evaluated (RNA‐seq analysis). The outcomes of this study indicated that increasing AURKA level was associated with the increase in the expression level of genes involved in the cell cycle (Figure 3D). These data recommended that *AURKA* plays a vital role in the growth and division of cancer cells. The mutation data for all 13 common cancers indicated that the *AURKA* gene had a variety of mutations in various cancer samples, and the percentage of SNP mutations was more than other types (Figure 3E). Examination of mutations showed that most of the SNPs in this gene were new, and none of which was more abundant. These results also indicated that *AURKA* might play a role in cancer cells, cell proliferation, and mutation in cancer.

### 3.3 The expression level of *AURKA* as a prognostic biomarker in some cancer types

**FIGURE 4:**
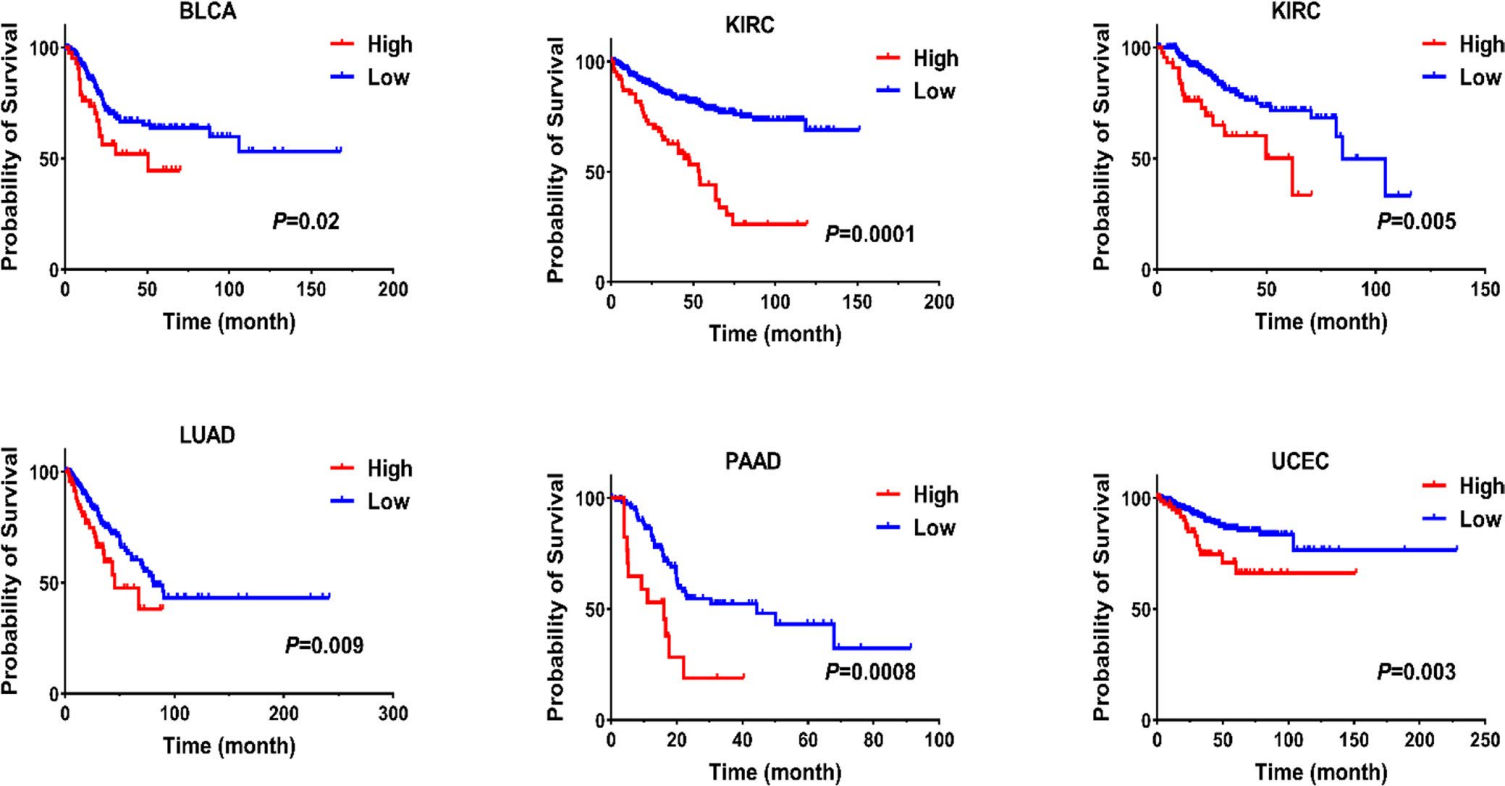
Increased *AURKA* expression is associated with poor survival in some cancers. The Kaplan–Meier for the effect of *AURKA* expression on the survival of patients with various cancers is shown based on clinical TCGA data. The scale data (*z*‐score) for each patient were used for analysis, and patients whose *AURKA* gene expression was one unit higher than the mean were considered as a high expression group

The expression matrix of each cancer and the clinical information of each patient were utilized to analyze the relation between the level of *AURKA* and prognosis in prevalent cancers. The cox regression test outcomes (Table 3) indicated that increased expression of *AURKA* gene was associated with a poor prognosis in the bladder (BLCA), kidney (KIRC), liver (LIHC), lung (LUAD), prostate (PAAD), and uterine cancer (UCEC). Kaplan–Meier analysis was used to confirm the results of Table 3, in these cancers, it also showed that the increased levels of the *AURKA* gene expression were associated with a poor prognosis in these cancers (Figure 4). These data proposed that the *AURKA* gene could be an excellent prognostic biomarker in a number of cancers.

**TABLE 3:** The cox regression test results between AURKA expression and patient survival

Cancer name	Beta	Hazard ratio (HR)	Log‐rank	HR lower––HR upper
Bladder urothelial carcinoma (BLCA)	0.30	1.36	0.018	1.12–1.76
Kidney renal clear cell carcinoma (KIRC)	0.57	1.76	0.000001	1.51–2.07
Liver hepatocellular carcinoma (LIHC)	0.34	1.41	0.008	1.1–1.82
Lung adenocarcinoma (LUAD)	0.32	1.38	0.001	1.13–1.7
Pancreatic adenocarcinoma (PAAD)	0.44	1.56	0.0007	1.21–2.02
Uterine corpus endometrial carcinoma (UCEC)	0.47	1.6	0.001	1.19–2.15

### 3.4 Association of *AURKA* expression level with drug resistance and sensitivity based on in silico study

**FIGURE 5:**
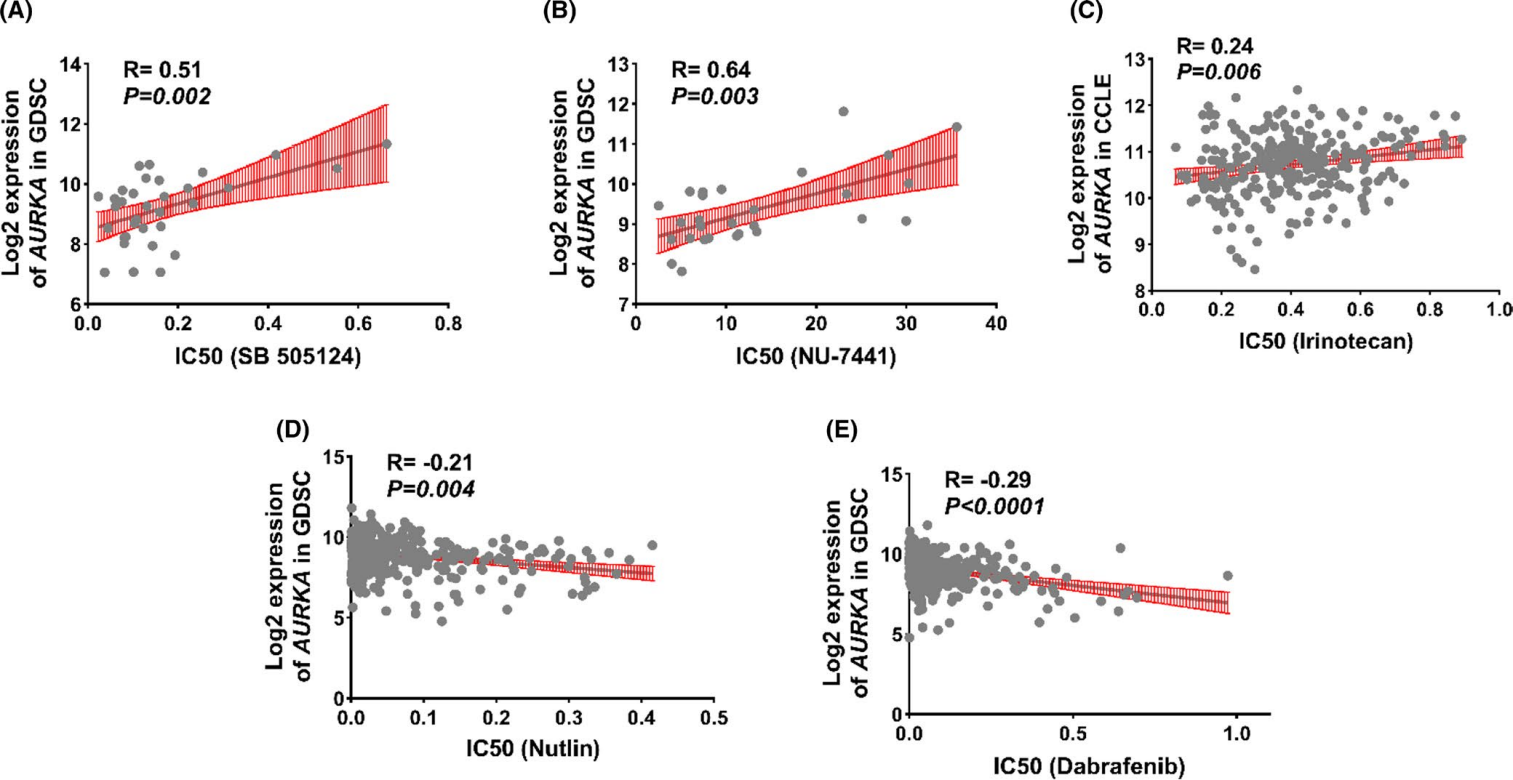
The *AURKA* level can play a role in drug resistance and sensitivity. The correlation test between *AURKA* expression and IC50 level for each drug in different cancer cell lines is shown. Data were extracted based on CCLE and GDSC databases

In order to obtain a proper assessment, the role of *AURKA* expression in drug resistance and sensitivity, GDSC and CCLE data were used (Section 2). Our results indicated that, the high level of *AURKA* has a correlation with increasing drug resistance to SB 505124, NU‐7441, and irinotecan drugs (Table 4). On the other hand, increasing the expression of *AURKA* can lead to sensitivity to dabrafenib and nutlin drugs (Table 4). To make sure of these results and visualizing, expression data and IC50 levels of all available drugs for all cell lines were extracted from GDSC and CCLE databases. Then Pearson correlation test was utilized between the *AURKA* expression level and IC50 of the mentioned drugs. Interestingly, previous results were also confirmed using this method as shown in Figure 5A–E (*p* < 0.01). The transcription level of *AURKA* can participate in resistance and sensitivity to some common chemotherapy drugs.

**TABLE 4:** Results of resistance and sensitivity in relation to AURKA expression based on PharmacoDB package analysis

Source	Drug name	Estimate	Degrees of freedom	FDR
GDSC	SB 505124	0.38	32	0.02
	NU‐7441	0.57	28	0.0008
	Dabrafenib	−0.18	487	0.0004
	Nutlin	−0.19	493	0.0003
CCLE	Irinotecan	0.17	286	0.01

### 3.5 Reduction of *AURKA* expression in presence of JQ1, Actinomycin D, and Camptothecin in some types of cell cancers with a poor prognosis for this gene

**FIGURE 6:**
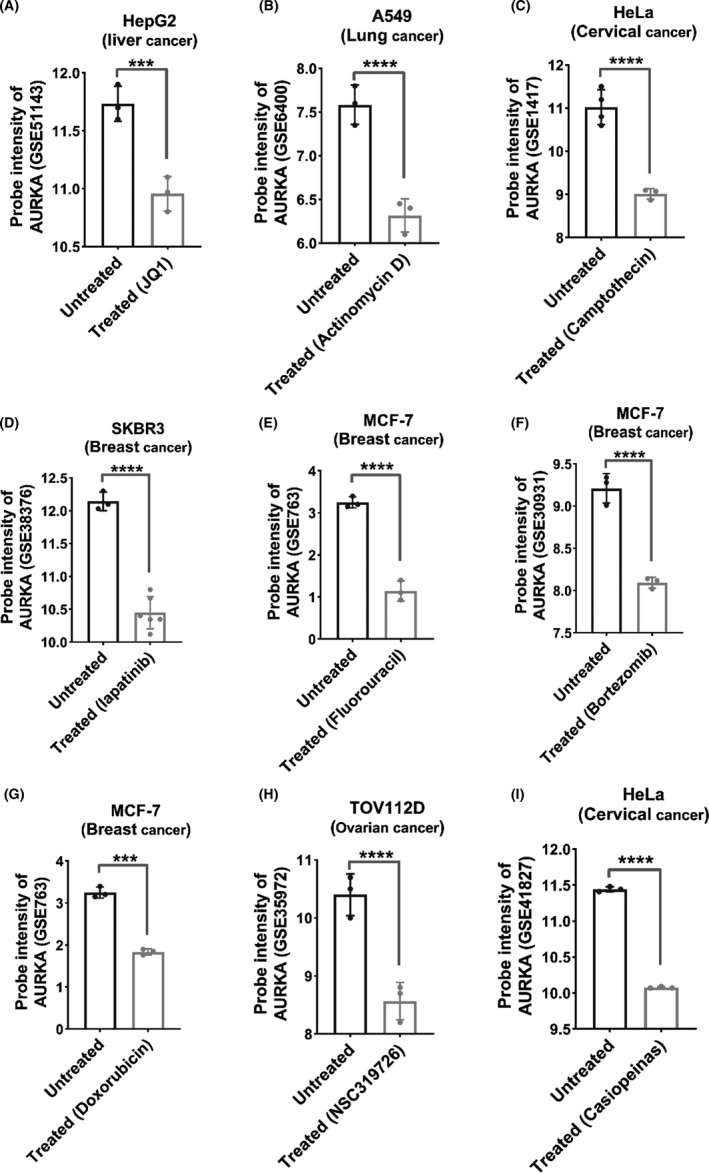
Some common chemotherapy drugs can directly or indirectly reduce *AURKA* expression. Using the information in the GEO, drugs that can reduce the level of *AURKA* are shown. All data are in logarithmic scale (****p < 0.0001, ***p < 0.001)

The data in the GEO database were used to assess drug effects on *AURKA* level and lead to *AURKA* downregulation. This analysis showed that JQ1, actinomycin D, and camptothecin had the greatest effect on the decreasing expression in the liver, lung, and uterine cancer cell lines, respectively (Figure 6A–C, *p* < 0.001). Lapatinib also had a marked influence on reducing the *AURKA* expression in the breast cancer cell line compared to other drugs (Figure 6D–G, *p* < 0.001). Also, the results of other drugs on different cell lines were shown (Figure 6H and I, *p* < 0.001). Moreover, all genes that showed altered expression in the presence of drugs were summarized in Table S1, briefly. Our findings mentioned that these drugs could be good candidates to lower the expression level of the *AURKA* gene in various cancer samples.

## 4 DISCUSSION

One of the most important elements in cancer studies is changing the level of genes expression in many types of cancers. Having a crucial role in the normal cells, some key genes altered by many items like a mutation that helps cancer cells growth. As some studies have shown that the expression level of the *AURKA* gene increased in many cancer cells including stomach, liver, colorectal, ovarian, breast, lung, bladder, head and neck, and prostate cancers.^32^, ^33^ In fact, what was achieved from our result was that the expression level of *AURKA* could be upregulated in stomach, colorectal, liver, ovarian, bladder, prostate, head and neck, lung, kidney, and lung cancers. Our data suggested that the high expression level of *AURKA* in all types of kidney cancers might be considered as a carcinogenesis factor that could inhibit apoptosis function. Therefore, the *AURKA* gene contributes in mitotic spindle formation, aneuploidy, and malignant transformation the high level of which could be strongly associated with carcinogenesis and cancer development.^1^, ^34^, ^35^

Based on a previous study, the high level of *AURKA* has been associated with clinical information such as tumor stage, lymph node, but not with gender, age, and race.^36^ Therefore, the aberrant level of *AURKA* could be mediating the molecular mechanisms underlying tumorigenesis.^33^, ^36^ *AURKA* might operate as an oncogene during the tumor progression by activating centrosome amplification and genomic instability.^7^, ^37^ Having a role in cell cycle, AURKA polymorphisms have a significant effect on cancers.^33^, ^38^ Although many *AURKA* polymorphisms were reported in previous studies, just some of which might indicate profound relation between these mutations and cancers.^38^, ^39^ Meanwhile, other recent studies showed that *AURKA* gene polymorphisms have a complete connection with increasing the risk of some cancers like liver and breast.^40^ For instance, previous studies showed that Polymorphism rs1047972 in the *AURKA* gene raises the level of liver cancer risk in spite of not having significant relation with bladder cancer risk.^24^, ^38^, ^41^ Besides, our bioinformatics data showed that most mutations in this gene are categorized as SNPs that are likely to be new. In conclusion, our pan‐cancer outcomes suggested that some new unknown mutations in *AURKA* could be discovered in many types of cancers.

*AURKA* gene has an important role in G2/M phase of cell division, and p53 is a key gene in suppressing tumors.^42^ In normal cells, *AURKA’s* function is to switch p53 off by phosphorylating Ser215 and Ser315,^42^ and it can inactivate p73 pro‐apoptotic functions.^43^, ^44^ Although deactivating tumor suppressors is a part of usual *AURKA* duties, it could raise cancer cells by permitting them to evade apoptosis.^42^, ^43^ *AURKA* overexpression might participate in progressing cancers by blocking DNA damage repair.^45^ As a result, this could be considered as an inverse connection between the high abnormal level of the *AURKA* gene and the low level of tumor suppressor proteins such as p53 and p71 that in this case, both of which are no longer able to induce apoptosis. In addition, our results indicated that the expression level of *AURKA* has correlation with the increasing expression level of genes that function in cell cycle. In doing so, based on our outcomes and other studies data, the *AURKA* gene probably has an effect on progressing and developing a cancer cell.

Furthermore, recent reports have mentioned that *AURKA* might have the potential to be considered as a therapeutic target in many types of cancers.^46^, ^47^ Some valuable AKIs have been identified for cancer therapy both in vitro and in vivo.^23^, ^48^ For instance, MLN8237 has been indicated that it could inhibit cell proliferation by damaging mitosis.^49^, ^50^ In fact, it makes autophagy and cell cycle arrest active and induce cell apoptosis.^50^ The high expression of *AURKA* could activate oncogenic factors such as c‐MYC, NF‐kB, and β‐catenin.^15^ In normal cells, c‐Myc expression can induce apoptosis indirectly, and on the contrary, the high level of c‐Myc in aberrant cells like uterine cancer could inhibit apoptosis and develop cancer cells.^51^, ^52^, ^53^ Recent studies indicated that some types of c‐Myc inhibitors like JQ1 could succeed in stopping cell proliferation in some types of cancers such as lung and uterine.^54^, ^55^ Therefore, the c‐Myc inhibitors could reduce the level of *AURKA* in cancers, and c‐Myc might be considered as a therapeutic target.^56^, ^57^ Based on our bioinformatics data, JQ1, actinomycin D1, camptothecin drugs decreased the expression level of *AURKA* in some kinds of cancers. Furthermore, lapatinib was able to lower the level of *AURKA* in breast cancer. Eventually, our outcomes showed that some drugs might be able to lower the level of *AURKA* in various cancers.

In addition to what has been mentioned about the expression level of *AURKA* whose overexpression could raise drug resistance and has a correlation with poor prognosis.^58^ In fact, our results showed that the high level of *AURKA* might have an influence on the overall survival of many types of cancer patients like breast, kidney, liver, lung, prostate, and uterine.^59^, ^60^ As some studies mentioned the overexpression of this gene has an inverse correlation with survival such as lung and bladder cancers.^36^ Recent studies showed that, the level of *AURKA* could be an independent prognostic factor for some types of cancers.^61^ Previous studies indicated that the high level of this gene was vital for tumorigenic capacity and drug resistance in some cancers like breast, lung, and stomach, the way our data showed the connection between the level of *AURKA* and drug resistance.^42^, ^62^ Our results indicated that the high level of *AURKA* has a correlation with increasing resistance to SB 505124, NU‐7441, irinotecan drugs, and meanwhile, this high expression has been associated with sensitivity to dabrafenib and nutlin. In doing so, these outcomes suggest that the relation between these drug resistance or sensitivity and the level of *AURKA* could be considered as an effective item to improve chemotherapy. Of course, this suggestion requires laboratory approval using cancer cell lines (high *AURKA* level vs low *AURKA* level) under commercial available molecules treatment.

## 5 CONCLUSION

Since the *AURKA* participates in the cell cycle and some signaling pathways in normal cells, the aberrant level of this gene can play an important role in many types of cancers. The abnormal expression level of *AURKA* correlates with some factors which could develop cancer cells. Although the high level of *AURKA* could be approved as a biomarker, it might make cancer patients show poor survival.

## CONFLICT OF INTEREST

All authors have declared that no competing interests exist.

## AUTHOR CONTRIBUTIONS

The design, sample collection, and conceptualization of study and methodology were performed by N.M. and K.G. Data mining, formal analysis, and investigation were performed by N.M. Supervision, validation, and visualization were performed by M.P. and K.G. Interpretation of the obtained information was performed by M.P. The manuscript was written by N.M. Review, editing, and approval of the manuscript were performed by M.P., K.G, and M.S. All authors read and approved the final manuscript.

## Supporting information

Figure S1

Table S1

## References

[1] Chen C, Song G, Xiang J, Zhang H, Zhao S, Zhan Y. AURKA promotes cancer metastasis by regulating epithelial‐mesenchymal transition and cancer stem cell properties in hepatocellular carcinoma. Biochem Biophys Res Commun. 2017;486(2):514‐520. 28322787 10.1016/j.bbrc.2017.03.075

[2] Siegel RL, Miller KD, Goding Sauer A, et al. Colorectal cancer statistics, 2020. CA A Cancer J Clin. 2020;70(3):145‐164 10.3322/caac.21601 32133645

[3] Sung H, Ferlay J, Siegel RL, et al. Global cancer statistics 2020: GLOBOCAN estimates of incidence and mortality worldwide for 36 cancers in 185 countries. CA Cancer J Clin. 2021;71(3):209‐249. 33538338 10.3322/caac.21660

[4] Jung E, Osswald M, Ratliff M, et al. Tumor cell plasticity, heterogeneity, and resistance in crucial microenvironmental niches in glioma. Nat Commun. 2021;12(1):1‐16. 33579922 10.1038/s41467-021-21117-3 PMC7881116

[5] Cui H, Shan H, Miao MZ, et al. Identification of the key genes and pathways involved in the tumorigenesis and prognosis of kidney renal clear cell carcinoma. Sci Rep. 2020;10(1):1‐10. 32144299 10.1038/s41598-020-61162-4 PMC7060270

[6] Dong P, Yu B, Pan L, Tian X, Liu F. Identification of key genes and pathways in triple‐negative breast cancer by integrated bioinformatics analysis. BioMed Res Int. 2018;2018:1‐10. 10.1155/2018/2760918 PMC6098886 30175120

[7] Goldenson B, Crispino JD. The aurora kinases in cell cycle and leukemia. Oncogene. 2015;34(5):537‐545. 24632603 10.1038/onc.2014.14 PMC4167158

[8] Gomaa A, Peng D, Chen Z, et al. Epigenetic regulation of AURKA by miR‐4715‐3p in upper gastrointestinal cancers. Sci Rep. 2019;9(1):1‐11. 31740746 10.1038/s41598-019-53174-6 PMC6861278

[9] Otto T, Sicinski P. Cell cycle proteins as promising targets in cancer therapy. Nat Rev Cancer. 2017;17(2):93. 28127048 10.1038/nrc.2016.138 PMC5345933

[10] Zheng F, Yue C, Li G, et al. Nuclear AURKA acquires kinase‐independent transactivating function to enhance breast cancer stem cell phenotype. Nat Commun. 2016;7(1):1‐17. 10.1038/ncomms10180 PMC4735655 26782714

[11] Dauch D, Rudalska R, Cossa G, et al. A MYC–aurora kinase A protein complex represents an actionable drug target in p53‐altered liver cancer. Nat Med. 2016;22(7):744‐753. 27213815 10.1038/nm.4107

[12] Fanale D, Bazan V, Corsini LR, et al. HIF‐1 is involved in the negative regulation of AURKA expression in breast cancer cell lines under hypoxic conditions. Breast Cancer Res Treat. 2013;140(3):505‐517. 23925655 10.1007/s10549-013-2649-0

[13] Huang C‐H, Chen C‐J, Chen P‐N, et al. Impacts of AURKA genetic polymorphism on urothelial cell carcinoma development. J Cancer. 2019;10(6):1370. 31031846 10.7150/jca.30014 PMC6485228

[14] Katsha A, Wang L, Arras J, et al. Activation of EIF4E by Aurora kinase A depicts a novel druggable axis in everolimus‐resistant cancer cells. Clin Cancer Res. 2017;23(14):3756‐3768. 28073841 10.1158/1078-0432.CCR-16-2141 PMC5503809

[15] Wang L, Arras J, Katsha A, et al. Cisplatin‐resistant cancer cells are sensitive to Aurora kinase A inhibition by alisertib. Mol Oncol. 2017;11(8):981‐995. 28417568 10.1002/1878-0261.12066 PMC5537695

[16] Liu Q, Kaneko S, Yang L, et al. Aurora‐A abrogation of p53 DNA binding and transactivation activity by phosphorylation of serine 215. J Biol Chem. 2004;279(50):52175‐52182. 15469940 10.1074/jbc.M406802200

[17] Liu X, Zhang Y, Wu S, et al. Palmatine induces G2/M phase arrest and mitochondrial‐associated pathway apoptosis in colon cancer cells by targeting AURKA. Biochem Pharmacol. 2020;175:113933. 32224138 10.1016/j.bcp.2020.113933

[18] Katayama H, Sasai K, Kawai H, et al. Phosphorylation by aurora kinase A induces Mdm2‐mediated destabilization and inhibition of p53. Nature Genet. 2004;36(1):55‐62. 14702041 10.1038/ng1279

[19] Puig‐Butille JA, Vinyals A, Ferreres JR, et al. AURKA overexpression is driven by FOXM1 and MAPK/ERK activation in melanoma cells harboring BRAF or NRAS mutations: impact on melanoma prognosis and therapy. J Invest Dermatol. 2017;137(6):1297‐1310. 28188776 10.1016/j.jid.2017.01.021

[20] de Martino M, Shariat SF, Hofbauer SL, et al. Aurora A kinase as a diagnostic urinary marker for urothelial bladder cancer. World J Urol. 2015;33(1):105‐110. 24562316 10.1007/s00345-014-1267-8

[21] Yang Y, Ding L, Zhou Q, et al. Silencing of AURKA augments the antitumor efficacy of the AURKA inhibitor MLN8237 on neuroblastoma cells. Cancer Cell Int. 2020;20(1):1‐16. 31920463 10.1186/s12935-019-1072-y PMC6947931

[22] Wang‐Bishop L, Chen Z, Gomaa A, et al. Inhibition of AURKA reduces proliferation and survival of gastrointestinal cancer cells with activated KRAS by preventing activation of RPS6KB1. Gastroenterology. 2019;156(3):662–675.e7. 30342037 10.1053/j.gastro.2018.10.030 PMC6368861

[23] Du R, Huang C, Liu K, Li X, Dong Z. Targeting AURKA in cancer: molecular mechanisms and opportunities for cancer therapy. Mol Cancer. 2021;20(1):1‐27. 33451333 10.1186/s12943-020-01305-3 PMC7809767

[24] Necchi A, Pintarelli G, Raggi D, Giannatempo P, Colombo F. Association of an aurora kinase A (AURKA) gene polymorphism with progression‐free survival in patients with advanced urothelial carcinoma treated with the selective aurora kinase A inhibitor alisertib. Invest New Drugs. 2017;35(4):524‐528. 28155045 10.1007/s10637-017-0440-5

[25] Mobley A, Zhang S, Bondaruk J, et al. Aurora kinase A is a biomarker for bladder cancer detection and contributes to its aggressive behavior. Sci Rep. 2017;7(1):1‐13. 28102366 10.1038/srep40714 PMC5244380

[26] Colaprico A, Silva TC, Olsen C, et al. TCGAbiolinks: an R/Bioconductor package for integrative analysis of TCGA data. Nucleic Acids Res. 2016;44(8):e71 26704973 10.1093/nar/gkv1507 PMC4856967

[27] Law CW, Chen Y, Shi W, Smyth GK. voom: precision weights unlock linear model analysis tools for RNA‐seq read counts. Genome Biol. 2014;15(2):1‐17. 10.1186/gb-2014-15-2-r29 PMC4053721 24485249

[28] Cibulskis K, Lawrence MS, Carter SL, et al. Sensitive detection of somatic point mutations in impure and heterogeneous cancer samples. Nature Biotechnol. 2013;31(3):213‐219. 23396013 10.1038/nbt.2514 PMC3833702

[29] Mayakonda A, Lin D‐C, Assenov Y, Plass C, Koeffler HP. Maftools: efficient and comprehensive analysis of somatic variants in cancer. Genome Res. 2018;28(11):1747‐1756. 30341162 10.1101/gr.239244.118 PMC6211645

[30] Leek JT, Johnson WE, Parker HS, Jaffe AE, Storey JD. The sva package for removing batch effects and other unwanted variation in high‐throughput experiments. Bioinformatics. 2012;28(6):882‐883. 22257669 10.1093/bioinformatics/bts034 PMC3307112

[31] Schmittgen TD, Livak KJ. Analyzing real‐time PCR data by the comparative CT method. Nat Protocols. 2008;3(6):1101. 18546601 10.1038/nprot.2008.73

[32] Treekitkarnmongkol W, Katayama H, Kai K, et al. Aurora kinase‐A overexpression in mouse mammary epithelium induces mammary adenocarcinomas harboring genetic alterations shared with human breast cancer. Carcinogenesis. 2016;37(12):1180‐1189. 27624071 10.1093/carcin/bgw097 PMC5137261

[33] Chou C‐H, Chou Y‐E, Chuang C‐Y, Yang S‐F, Lin C‐W. Combined effect of genetic polymorphisms of AURKA and environmental factors on oral cancer development in Taiwan. PLoS ONE. 2017;12(2):e0171583. 28152093 10.1371/journal.pone.0171583 PMC5289639

[34] Goos JA, Coupé VM, Diosdado B, et al. Aurora kinase A (AURKA) expression in colorectal cancer liver metastasis is associated with poor prognosis. Brit J Cancer. 2013;109(9):2445‐2452. 24104968 10.1038/bjc.2013.608 PMC3817339

[35] Blanco I, Kuchenbaecker K, Cuadras D, et al. Assessing associations between the AURKA‐HMMR‐TPX2‐TUBG1 functional module and breast cancer risk in BRCA1/2 mutation carriers. PLoS ONE. 2015;10(4):e0120020. 25830658 10.1371/journal.pone.0120020 PMC4382299

[36] Kamran M, Long Z, Xu D, et al. Aurora kinase A regulates Survivin stability through targeting FBXL7 in gastric cancer drug resistance and prognosis. Oncogenesis. 2017;6(2):e298. 28218735 10.1038/oncsis.2016.80 PMC5337621

[37] Chuang T‐P, Wang J‐Y, Jao S‐W, et al. Over‐expression of AURKA, SKA3 and DSN1 contributes to colorectal adenoma to carcinoma progression. Oncotarget. 2016;7(29):45803. 27329586 10.18632/oncotarget.9960 PMC5216762

[38] Dai Z‐J, Kang H‐F, Wang X‐J, et al. Association between genetic polymorphisms in AURKA (rs2273535 and rs1047972) and breast cancer risk: a meta‐analysis involving 37,221 subjects. Cancer Cell Int. 2014;14(1):1‐8. 25253995 10.1186/s12935-014-0091-y PMC4173109

[39] Lee C‐P, Chiang S‐L, Lee C‐H, et al. AURKA Phe31Ile polymorphism interacted with use of alcohol, betel quid, and cigarettes at multiplicative risk of oral cancer occurrence. Clin Oral Invest. 2015;19(8):1825‐1832. 10.1007/s00784-015-1432-5 25697104

[40] Wang B, Hsu C‐J, Chou C‐H, et al. Variations in the AURKA gene: biomarkers for the development and progression of hepatocellular carcinoma. Int J Med Sci. 2018;15(2):170. 29333101 10.7150/ijms.22513 PMC5765730

[41] Mesic A, Rogar M, Hudler P, Juvan R, Komel R. Association of the AURKA and AURKC gene polymorphisms with an increased risk of gastric cancer. IUBMB Life. 2016;68(8):634‐644. 27270838 10.1002/iub.1521

[42] Sehdev V, Katsha A, Arras J, et al. HDM2 regulation by AURKA promotes cell survival in gastric cancer. Clin Cancer Res. 2014;20(1):76‐86. 24240108 10.1158/1078-0432.CCR-13-1187 PMC3947328

[43] Vilgelm AE, Pawlikowski JS, Liu Y, et al. Mdm2 and aurora kinase a inhibitors synergize to block melanoma growth by driving apoptosis and immune clearance of tumor cells. Cancer Res. 2015;75(1):181‐193. 25398437 10.1158/0008-5472.CAN-14-2405 PMC4286469

[44] Hsueh K‐W, Fu S‐L, Chang C‐B, Chang Y‐L, Lin C‐H. A novel Aurora‐A‐mediated phosphorylation of p53 inhibits its interaction with MDM2. Biochim Biophys Acta Proteins Proteom. 2013;1834(2):508‐515. 10.1016/j.bbapap.2012.11.005 23201157

[45] Katayama H, Wang J, Treekitkarnmongkol W, et al. Aurora kinase‐A inactivates DNA damage‐induced apoptosis and spindle assembly checkpoint response functions of p73. Cancer Cell. 2012;21(2):196‐211. 22340593 10.1016/j.ccr.2011.12.025 PMC3760020

[46] Umene K, Yanokura M, Banno K, et al. Aurora kinase A has a significant role as a therapeutic target and clinical biomarker in endometrial cancer. Int J Oncol. 2015;46(4):1498‐1506. 25625960 10.3892/ijo.2015.2842 PMC4356503

[47] Guo M, Lu S, Huang H, et al. Increased AURKA promotes cell proliferation and predicts poor prognosis in bladder cancer. BMC Syst Biol. 2018;12(7):11‐17. 30547784 10.1186/s12918-018-0634-2 PMC6293497

[48] Caputo E, Miceli R, Motti ML, et al. AurkA inhibitors enhance the effects of B‐RAF and MEK inhibitors in melanoma treatment. J Transl Med. 2014;12(1):1‐9. 25074438 10.1186/s12967-014-0216-z PMC4237855

[49] Dees EC, Infante JR, Cohen RB, et al. Phase 1 study of MLN8054, a selective inhibitor of Aurora A kinase in patients with advanced solid tumors. Cancer Chemother Pharmacol. 2011;67(4):945‐954. 20607239 10.1007/s00280-010-1377-y PMC3026871

[50] Liu Y, Hawkins OE, Su Y, et al. Targeting aurora kinases limits tumour growth through DNA damage‐mediated senescence and blockade of NF‐κB impairs this drug‐induced senescence. EMBO Mol Med. 2013;5(1):149‐166. 23180582 10.1002/emmm.201201378 PMC3569660

[51] Soucek L, Evan GI. The ups and downs of Myc biology. Curr Opin Genet Dev. 2010;20(1):91‐95. 19962879 10.1016/j.gde.2009.11.001 PMC2822095

[52] Nair R, Roden D, Teo W, et al. c‐Myc and Her2 cooperate to drive a stem‐like phenotype with poor prognosis in breast cancer. Oncogene. 2014;33(30):3992‐4002. 24056965 10.1038/onc.2013.368

[53] Zhao Z‐N, Bai J‐X, Zhou Q, et al. TSA suppresses miR‐106b‐93‐25 cluster expression through downregulation of MYC and inhibits proliferation and induces apoptosis in human EMC. PLoS ONE. 2012;7(9):e45133. 23028803 10.1371/journal.pone.0045133 PMC3446970

[54] Albihn A, Johnsen JI, Henriksson MA. MYC in oncogenesis and as a target for cancer therapies. Adv Cancer Res. 2010;107:163‐224. 20399964 10.1016/S0065-230X(10)07006-5

[55] Fletcher S, Prochownik EV. Small‐molecule inhibitors of the Myc oncoprotein. BBA‐Gene Regul Mech. 2015;1849(5):525‐543. 10.1016/j.bbagrm.2014.03.005 PMC4169356 24657798

[56] Filippakopoulos P, Qi J, Picaud S, et al. Selective inhibition of BET bromodomains. Nature. 2010;468(7327):1067‐1073. 20871596 10.1038/nature09504 PMC3010259

[57] Qiu H, Li J, Clark LH, et al. JQ1 suppresses tumor growth via PTEN/PI3K/AKT pathway in endometrial cancer. Oncotarget. 2016;7(41):66809. 27572308 10.18632/oncotarget.11631 PMC5341839

[58] Komoto TT, Bernardes TM, Mesquita TB, et al. Chalcones repressed the AURKA and MDR proteins involved in metastasis and multiple drug resistance in breast cancer cell lines. Molecules. 2018;23(8):2018. 10.3390/molecules23082018 PMC6222917 30104527

[59] Galusic D, Lucijanic M, Livun A, et al. Higher AURKA and PLK1 expression are associated with inferior overall survival in patients with myelofibrosis. Blood Cells Mol Dis. 2020;81:102396. 10.1016/j.bcmd.2019.102396 31837568

[60] Lee JW, Parameswaran J, Sandoval‐Schaefer T, et al. Combined aurora kinase A (AURKA) and WEE1 inhibition demonstrates synergistic antitumor effect in squamous cell carcinoma of the head and neck. Clin Cancer Res. 2019;25(11):3430‐3442. 30755439 10.1158/1078-0432.CCR-18-0440 PMC6548643

[61] Al‐Khafaji AS, Marcus MW, Davies M, et al. AURKA mRNA expression is an independent predictor of poor prognosis in patients with non‐small cell lung cancer. Oncol Lett. 2017;13(6):4463‐4468. 28588715 10.3892/ol.2017.6012 PMC5452881

[62] Zhang Y, Chen H‐X, Zhou S‐Y, et al. Sp1 and c‐Myc modulate drug resistance of leukemia stem cells by regulating survivin expression through the ERK‐MSK MAPK signaling pathway. Mol Cancer. 2015;14(1):1‐18. 25890196 10.1186/s12943-015-0326-0 PMC4357193

