# Supplementary figures and images for: Integrated pan‐cancer of AURKA expression and drug sensitivity analysis reveals increased expression of AURKA is responsible for drug resistance
Source: Cancer Med. 2021 Aug 1;10(18):6428–41. doi: 10.1002/cam4.4161 (PMC8446408; doi:10.1002/cam4.4161)

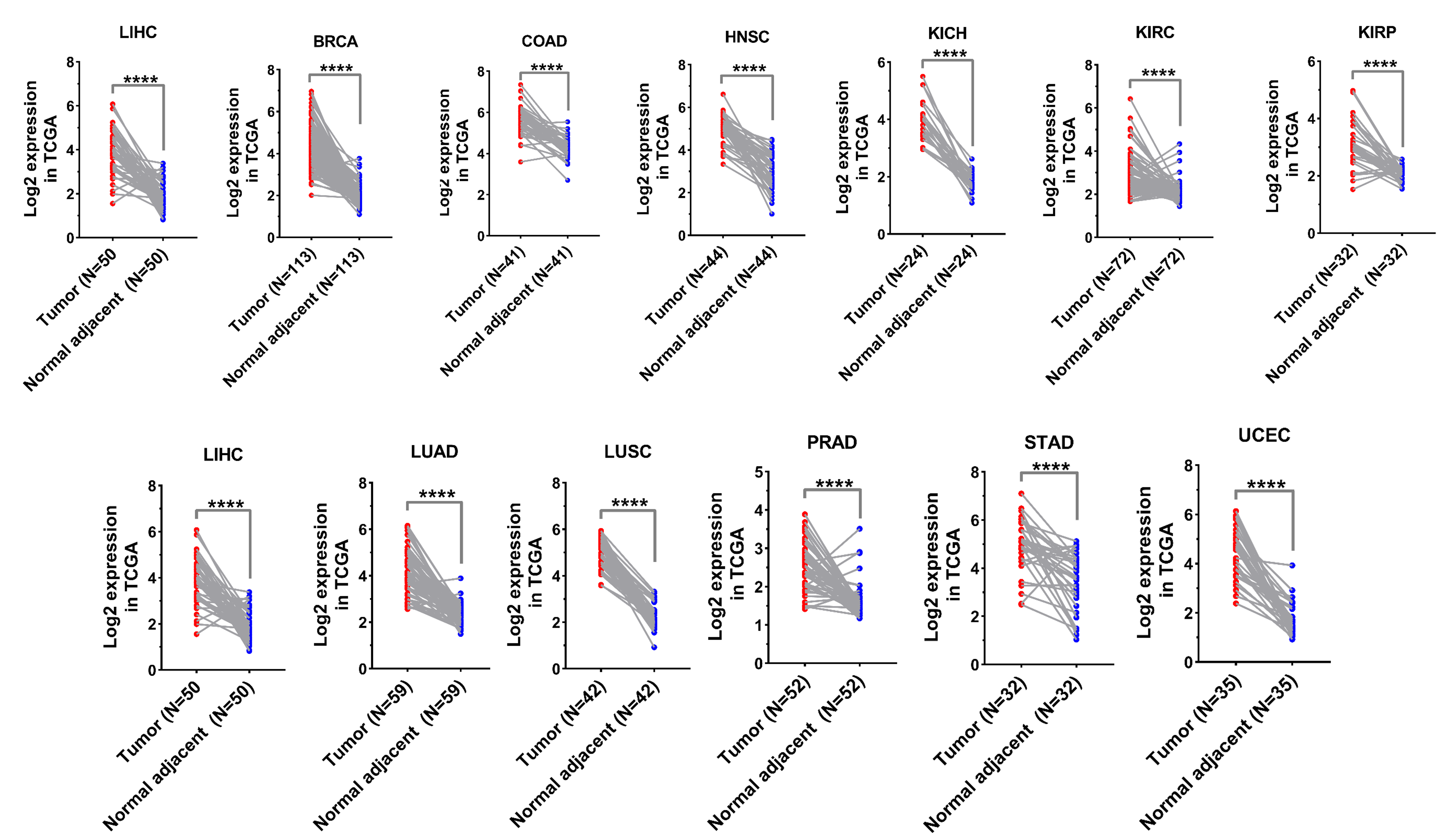

Supplement: Supplementary file 1 — Figure S1 [file CAM4-10-6428-s001.tif]
